# Factors associated with interval colorectal cancer after negative FIT: Results of two screening rounds in the Dutch FIT‐based CRC screening program

**DOI:** 10.1002/ijc.34373

**Published:** 2022-12-01

**Authors:** Emilie C. H. Breekveldt, Esther Toes‐Zoutendijk, Hilliene J. van de Schootbrugge‐Vandermeer, Lucie de Jonge, Arthur I. Kooyker, Manon C. W. Spaander, Anneke J. van Vuuren, Folkert J. van Kemenade, Christian Ramakers, Evelien Dekker, Iris D. Nagtegaal, Monique E. van Leerdam, Iris Lansdorp‐Vogelaar

**Affiliations:** ^1^ Department of Public Health Erasmus MC University Medical Center Rotterdam The Netherlands; ^2^ Department of Gastroenterology and Hepatology Netherlands Cancer Institute—Antoni van Leeuwenhoek Hospital Amsterdam The Netherlands; ^3^ Department of Gastroenterology and Hepatology Erasmus MC University Medical Center Rotterdam The Netherlands; ^4^ Department of Pathology Erasmus MC University Medical Center Rotterdam The Netherlands; ^5^ Department of Clinical Chemistry Erasmus MC University Medical Center Rotterdam The Netherlands; ^6^ Department of Gastroenterology and Hepatology Amsterdam University Medical Centers—Academic Medical Center Amsterdam The Netherlands; ^7^ Department of Pathology Radboud University Medical Center Nijmegen The Netherlands; ^8^ Department of Gastroenterology and Hepatology Leiden University Medical Center Leiden the Netherlands

**Keywords:** colorectal cancer, colorectal cancer screening, fecal immunochemical testing

## Abstract

The interval colorectal cancer (CRC) rate after negative fecal immunochemical testing (FIT) is an important quality indicator of CRC screening programs. We analyzed the outcomes of two rounds of the FIT‐based CRC screening program in the Netherlands, using data from individuals who participated in FIT‐screening from 2014 to 2017. Data of individuals with one prior negative FIT (first round) or two prior negative FITs (first and second round) were included. Outcomes included the incidence of interval CRC in FIT‐negative participants (<47 μg Hb/g feces [μg/g]), FIT‐sensitivity, and the probability of detecting an interval CRC by fecal hemoglobin concentration (f‐Hb). FIT‐sensitivity was estimated using the detection method and the proportional incidence method (based on expected CRC incidence). Logistic regression analysis was performed to estimate whether f‐Hb affects probability of detecting interval CRC, adjusted for sex‐ and age‐differences. Incidence of interval CRC was 10.4 per 10 000 participants after the first and 9.6 after the second screening round. FIT‐sensitivity based on the detection method was 84.4% (95%CI 83.8‐85.0) in the first and 73.5% (95% CI 71.8‐75.2) in the second screening round. The proportional incidence method resulted in a FIT‐sensitivity of 76.4% (95%CI 73.3‐79.6) in the first and 79.1% (95%CI 73.7‐85.3) in the second screening round. After one negative FIT, participants with f‐Hb just below the cut‐off (>40‐46.9 μg/g) had a higher probability of detecting an interval CRC (OR 16.9; 95%CI: 14.0‐20.4) than had participants with unmeasurable f‐Hb (0‐2.6 μg/g). After two screening rounds, the odds ratio for interval CRC was 12.0 (95%CI: 7.8‐17.6) for participants with f‐Hb just below the cut‐off compared with participants with unmeasurable f‐Hb. After both screening rounds, the Dutch CRC screening program had a low incidence of interval CRC and an associated high FIT‐sensitivity. Our findings suggest there is a potential for further optimizing CRC screening programs with the use of risk‐stratified CRC screening based on prior f‐Hb.

## Graphical abstract

What's new?

The sensitivity of the fecal immunochemical test for occult human hemoglobin and its associated interval cancer rate are an important quality indicator of colorectal cancer screening programs. This study shows that, after two screening rounds, the Dutch colorectal cancer screening program has a high FIT‐sensitivity with a low incidence of interval colorectal cancer. The probability of detecting interval colorectal cancer however increased as fecal hemoglobin concentrations rose below the cut‐off value. The findings suggest there is potential for further optimizing colorectal cancer screening programs through the implementation of risk‐stratified colorectal cancer screening based on prior fecal hemoglobin concentrations.

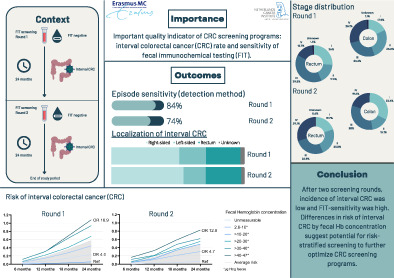

**Abbreviations**
AIC: akaike information criterion
AN: advanced neoplasia
CI: confidence interval
CRC: colorectal cancer
f‐Hb: fecal hemoglobin
FIT: fecal immunochemical test
IC: interval colorectal cancer
IQR: interquartile range
NCR: Netherlands Cancer Registry
NOS: not otherwise specified
OR: odds ratio
PI: proportional incidence
RIVM: National Institute for Public Health and the Environment (RIVM)
RR: rate ratio
SD‐CRC: screening‐detected colorectal cancer
TNM: tumor, node, metastases

## 1 INTRODUCTION

Organized colorectal cancer (CRC) screening programs have been adopted widely with the aim to reduce CRC‐related mortality. These programs are mostly based on fecal immunochemical testing for occult human hemoglobin (FIT). The quantitative nature of FIT (μg Hb/g feces) allows for adjusting the cut‐off for a positive test result. Several factors can be considered to determine the optimal cut‐off; that is, positivity rate, colonoscopy capacity and sensitivity of FIT for CRC.

The incidence of interval CRCs after a negative FIT may serve to indicate the sensitivity of FIT, based on the occurrence of false‐negative FITs. Evaluation of the sensitivity of FIT and the incidence of interval CRC is necessary to assess the quality of the program.^1^ Besides, it can reveal information on characteristics of interval CRCs that might provide insight on the number of cancers missed in FIT‐based screening. Previous research showed that higher fecal Hb (f‐Hb) concentrations in prior screening rounds were associated with higher detection of CRC or advanced neoplasia (AN) in subsequent screening rounds, as well as a higher probability of detecting interval CRC after negative FIT.^2^, ^3^, ^4^, ^5^, ^6^, ^7^, ^8^ Still, the small sample sizes in those studies call for validation of this risk factor in larger populations.

In the Netherlands, an organized FIT‐based screening program went ahead in 2014, inviting all individuals eligible for screening every two years. The complete target population has been invited from 2019 onwards and participation rates are consistently high (around 72%). A previous study from our group found that the Dutch CRC screening program revealed a low incidence of interval CRC and an associated high sensitivity of FIT after one screening round.^5^ Only few studies are available on the incidence of interval CRC and sensitivity after multiple screening rounds, especially detailed data on specific screening rounds are scarce.^9^

In this study, we evaluated the incidence of interval CRC and sensitivity of FIT within the framework of the FIT‐based CRC screening program in the Netherlands, both after one screening round (one prior negative FIT) and after two screening rounds (two prior negative FITs). In addition, we assessed characteristics (ie, localization and stage distribution) of these interval CRCs, as well as the probability of detecting interval CRC based on f‐Hb concentrations at prior screening.

## 2 METHODS

### 2.1 Dutch national screening program

In 2014, the Dutch national CRC screening program was introduced, for which all individuals aged 55 to 75 were invited biennially for FIT‐based screening (FOB‐Gold, Sentinel Diagnostics, Milan, Italy). The program was gradually rolled out by birth cohort. Since 2019, all individuals in the target population (around 4.4 million) have been invited at least once. Those with a positive FIT were referred for colonoscopy; in case of a negative FIT, participants were invited for a second test 24 months later. Initially, a FIT positivity cut‐off of 15 μg Hb/g feces was used; this was adjusted to 47 μg Hb/g feces in June 2014. The rationale for this choice has been described previously.^10^

### 2.2 Data collection

Real‐time data from the Dutch CRC program stored in the national screening information system (ScreenIT) were linked with data from the Netherlands Cancer Registry (NCR). This would enable identifying CRCs diagnosed after a positive and after a negative FIT. Data from the NCR, including complete data on incidence and stage distribution, covered the period from January 1, 2014 to November 1, 2019. To ensure complete follow‐up for analyses on interval CRC (24 months), only participants tested between January 1, 2014 and November 1, 2017 were included in the analyses. To maintain homogeneity within groups, only participants tested at the positivity cut‐off of 47 μg Hb/g feces that was initiated in June 2014 were included. First screening round participants were defined as participants with one prior negative or positive FIT at the first invitation round. Second screening round participants were defined as participants with one prior negative FIT at the first invitation round and subsequent negative or positive FIT at the second invitation round.

### 2.3 Definitions

A negative FIT was defined as a FIT with f‐Hb concentration <47 μg Hb/g feces. A positive FIT was defined as a FIT with f‐Hb concentration ≥47 μg Hb/g feces. Interval CRC was defined as CRC diagnosed after a negative FIT and before invitation to the next screening round, according to the proposed nomenclature by the World Endoscopy Organization.^11^ For participants who were not eligible for the subsequent screening round because they had reached the upper age limit, interval CRC was defined as CRC diagnosed within 24 months after a negative FIT. Screening‐detected CRC was defined as CRC diagnosed within 180 days after a colonoscopy following a positive FIT. The episode sensitivity of FIT was defined as the percentage of individuals in the screened population who were identified by the FIT and confirmed as truly positive (ie, having CRC) at colonoscopy. Episode sensitivity reflects the full diagnostic process of CRC screening per screening round.^12^

Interval CRC was categorized as right‐sided (caecum to transverse colon, C18.0, C18.2‐C18.4), left‐sided (splenic flexure to rectosigmoid, C18.5‐C18.7, C19), rectum (C20), or overlapping and not otherwise specified (NOS; C18.8‐C18.9).^13^ Appendiceal cancers (C18.1) were excluded from analyses. In case of synchronous CRCs, the CRC with the most advanced stage was included in the analyses. Stage distribution was determined using the effective Tumor, Node, Metastases (TNM)‐classification at year of diagnosis (seventh edition in 2014‐2016, eighth edition from 2017).

### 2.4 Outcomes

Primary outcomes were the incidence of interval CRC, the episode sensitivity and the probability of detecting interval CRC by f‐Hb concentration after the first and second round, respectively. The incidence of interval CRC was calculated by dividing the number of interval CRCs by the total number of participants with a negative FIT in the same screening round, and is presented per 10 000 participants with a negative FIT. Furthermore, we determined the probability of detecting interval CRC by f‐Hb concentration, corrected for sex‐ and age‐differences. Secondary outcomes were localization and stage distribution of interval CRCs and screening‐detected CRCs diagnosed after the first and second round.

### 2.5 Statistical analysis

We estimated the incidence of interval CRC and episode sensitivity of FIT for CRC after the first and second screening round of the Dutch national CRC screening program. Episode sensitivity was estimated in two ways: through the detection method and the proportional incidence (PI) method. Episode sensitivity according to the detection method was calculated from the number of screening‐detected CRCs (SD‐CRC) per round divided by the sum of interval CRCs and screening‐detected CRCs for that specific round, using the formula: $\text{Sensitivity}\;\left(\text{detection method}\right)=\frac{\mathrm{SD}-\mathrm{CRC}}{\mathrm{IC}+\mathrm{SD}-\mathrm{CRC}}$. Episode sensitivity according to the PI method was calculated from the expected CRC incidence extrapolating data from the pre‐screening era. A log‐linear Poisson model served to estimate the expected CRC incidence from age‐specific CRC incidence trends in the Netherlands in the pre‐screening era (2009‐2013). Based on this estimate, the expected sex‐ and age‐specific CRC incidences for the first (2014‐2017) and second (2016‐2017) round were calculated. Trends were standardized by sex‐ and age distributions of the study population. Next, the proportional incidence or rate ratio (RR) of interval CRC (IC) was estimated as the number of interval CRCs divided by the length of the interval multiplied by the expected annual CRC incidence (E) for that specific sex‐ or age group, using the formula: $\mathrm{RR}=\frac{\mathrm{IC}}{\text{Interval length}\;\left(\text{years}\right)\times E}$. The mean interval length was 1.97 years (23.7 months) in the first round and 1.96 years (23.5 months) in the second round. The episode sensitivity was calculated using the formula: $\text{Sensitivity}\;\left(\mathrm{PI}\;\text{method}\right)=1-\mathrm{RR}$.

The incidence of interval CRC and the sensitivity of FIT are summarized using standard descriptive statistics, displaying the 95% confidence interval (CI).

Chi‐square testing was performed to compare localization and stage distribution of interval CRCs with screening‐detected CRCs after the first and second round, respectively. Calculated *p* values are two‐sided and are considered statistically significant when <.05.

Logistic regression analysis was performed to determine the odds ratio (OR) of interval CRC after the first and after the second round, based on f‐Hb concentration, adjusted for sex‐ and age‐differences. Only data of individuals who participated in both rounds were used to determine the number of interval CRCs after the second round. F‐Hb concentrations were categorized as: unmeasurable (0‐2.6 μg Hb/g feces; below limit of detection), >2.6 to 10 μg Hb/g feces, >10 to 20 μg Hb/g feces, >20 to 30 μg Hb/g feces, >30 to 40 μg Hb/g feces and >40 to 46.9 μg Hb/g feces. Five age categories were defined with respect to interval CRCs after the first round: 55‐59, 60‐64, 65‐69, 70‐74 and ≥ 75 years. Complete data on interval CRCs after the second round were available for only three age categories: namely 60‐64, 65‐69 and ≥70 years.

We evaluated the probability of detecting an interval CRC using multiple models. Model 1 concerned the OR of detecting interval CRC based on f‐Hb concentration of participants with a negative FIT at the first round. Model 2 concerned the OR of detecting interval CRC based on the last measured f‐Hb concentration of participants with a negative FIT at the second round. Lastly, f‐Hb concentrations at both the first *and* second round of participants with a negative FIT in both rounds were incorporated (Models 3a‐c). These models were variations of model 2. Model 3a included dichotomous (0‐2.6 vs >2.6‐46.9 μg Hb/g feces) f‐Hb concentrations of the first round as well as categorical f‐Hb concentrations of the second round. Model 3b included summed f‐Hb concentrations of both rounds, dividing this added value into quantiles. Model 3c included categorical f‐Hb concentrations of both rounds, as opposed to only the last f‐Hb concentration measured in the second round (Model 2). Goodness‐of‐fit of the models was determined by comparing Akaike Information Criterion (AIC) scores of the different models.

Data management and analysis were performed using R version 3.5.0 (R Foundation for Statistical Computing, Vienna, Austria).

## 3 RESULTS

**FIGURE 1:**
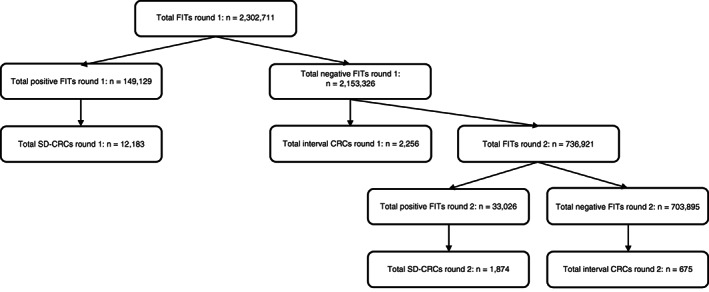
Flowchart displaying numbers for first and second round. CRC, colorectal cancer; FIT, fecal immunochemical test; SD‐CRC, screening‐detected colorectal cancer

The first round included 2 302 711 individuals of whom 2 153 582 (93.5%) had a negative FIT, and 2256 of the latter had been diagnosed with an interval CRC (Figure 1 and Table 1). Median age of the FIT‐negative participants was 67 years (interquartile range [IQR]: 63‐73). At the first round, 149 129 (6.5%) participants had a positive FIT, of whom 12 183 had been diagnosed with a screening‐detected CRC (Figure 1). Median age in FIT‐positive participants was 65 years (IQR: 61‐71). The incidence of interval CRCs in participants with a negative FIT was 10.4 per 10 000 (Table 2). The episode sensitivity of FIT was 84.4% (95%CI 83.8‐85.0) as determined with the detection method, and 76.4% (95%CI 73.3‐79.6) as determined with the PI method (Table 2 and Supplementary Table 1).

**TABLE 1:** Characteristics study population

	First screening round		Second screening round	
	Negative FIT, n (%)	Interval CRC, n (%)	Negative FIT, n (%)	Interval CRC, n (%)
Total	2 153 582	2256	703 895	675
Men	1 024 314 (47.6)	1178 (52.2)	334 559 (47.5)	366 (54.2)
Women	1 129 268 (52.4)	1078 (47.8)	369 336 (52.5)	309 (45.8)
Age distribution				
56‐59	336 917 (15.6)	122 (5.4)	—	—
60‐64	767 684 (35.6)	594 (26.3)	76 543 (10.9)	46 (6.8)
65‐69	626 627 (29.1)	729 (32.3)	532 388 (75.6)	519 (76.9)
70‐74	171 944 (8.0)	279 (12.4)	94 964 (13.5)	110 (16.3)
≥75	250 410 (11.6)	532 (23.6)	—	—
Prior f‐Hb concentration (μg Hb/g feces)				
Unmeasurable (0‐2.6)	1 907 528 (88.7)	1143 (50.7)	654 010 (92.9)	441 (65.3)
>2.6‐10	127 256 (5.9)	324 (14.3)	21 513 (3.1)	69 (10.2)
>10‐20	62 479 (2.9)	292 (12.9)	13 305 (1.9)	66 (9.8)
>20‐30	26 723 (1.2)	195 (8.6)	6895 (1.0)	39 (5.8)
>30‐40	18 603 (0.9)	181 (8.0)	5149 (0.7)	35 (5.2)
>40‐46.9	10 993 (0.5)	121 (5.4)	3023 (0.4)	25 (3.7)

Abbreviations: CRC, colorectal cancer; F‐Hb, fecal hemoglobin; FIT, fecal immunochemical testing.

**TABLE 2:** Incidence of interval CRC after negative FIT and sensitivity of FIT

	Number			Incidence rate/10000		CRC predicted^a^	RR	Sensitivity (detection method) (%, 95%CI)	Sensitivity (PI method) (%, 95%CI)
	Population screened	IC	SDC	IC	SDC		IC	SDC/SDC + IC	1‐RR
Round 1									
Sex									
Male	1 113 736	1178	7584	10.6	68.1	50.6	0.21	86.6 (85.8‐87.3)	79.0 (74.7‐83.7)
Female	1 188 975	1078	4599	9.1	38.7	33.1	0.28	81.0 (80.0‐82.0)	72.5 (68.3‐77.0)
Age (yrs)									
55‐59	353 178	122	899	3.5	25.5	17.4	0.20	88.1 (86.1‐90.0)	79.9 (66.9‐95.4)
60‐64	813 106	594	3248	7.3	40.0	29.5	0.25	84.5 (83.4‐85.7)	75.3 (69.4‐81.6)
65‐69	673 110	729	3985	10.8	59.2	46.1	0.23	84.5 (83.5‐85.6)	76.6 (71.2‐82.3)
70‐74	187 583	279	1511	14.9	80.6	62.9	0.24	84.4 (82.7‐86.1)	76.3 (67.9‐85.8)
≥75	275 734	532	2540	19.3	92.1	83.5	0.23	82.7 (81.3‐84.0)	76.9 (70.6‐83.7)
Total	2 302 711	2256	12 183	9.8	52.9	41.6	0.24	84.4 (83.8‐85.0)	76.4 (73.3‐79.6)
Round 2									
Sex									
Male	334 559	366	1066	10.9	31.9	56.6	0.19	74.4 (72.2‐76.7)	80.7 (72.9‐89.4)
Female	369 336	299	808	8.1	21.9	36.1	0.22	73.0 (70.4‐75.6)	77.5 (69.2‐86.8)
Age (yrs)									
60‐64	76 542	46	143	6.0	18.7	29.1	0.21	75.7 (69.5‐81.8)	79.4 (59.4‐106.0)
65‐69	532 388	519	1416	9.7	26.6	45.5	0.21	73.2 (71.2‐75.2)	78.7 (72.2‐85.7)
≥70	94 964	110	315	11.6	33.2	62.3	0.19	74.1 (70.0‐78.3)	81.4 (67.5‐98.1)
Total	703 895	675	1874	9.6	26.6	45.9	0.21	73.5 (71.8‐75.2)	79.1 (73.3‐85.3)

Abbreviations: CI, confidence interval; CRC, colorectal cancer; IC, interval colorectal cancer; PI, proportional incidence; RR, rate ratio; SDC, screening‐detected colorectal cancer; Yrs, years.

^a^ Based on expected CRC incidence using Poisson log linear regression to extrapolate CRC incidence data from the pre‐screening era. Displayed for the screening interval of 1.97 years in the first round and 1.96 years in the second round.

The second round included 736 921 individuals, of whom 703 895 (95.5%) had a negative FIT, and 675 of the latter had been diagnosed with an interval CRC (Figure 1 and Table 1). Median age of the FIT‐negative participants was 67 years (IQR: 66‐69). At the second round, 33 026 (4.5%) participants had a positive FIT, of whom 1874 had been diagnosed with a screening‐detected CRC (Figure 1). The median age of the FIT‐positive participants was 67 years (IQR: 65‐69). The incidence of interval CRC in participants with a negative FIT was 9.6 per 10 000 (Table 2). After the second round, the episode sensitivity of FIT was 73.5% (95%CI 71.8‐75.2) as determined with the detection method and 79.1% (73.3‐85.3) as determined with the PI method (Table 2 and Supplementary Table 2). The incidence of interval CRC after the first round was significantly higher than after the second round (*P* = .04). Furthermore, the incidence of interval CRC was significantly higher in men than in women in both the first (*P* = .003) and second (*P* = .002) round (Table 1).

### 3.1 Stage distribution and localization

**FIGURE 2:**
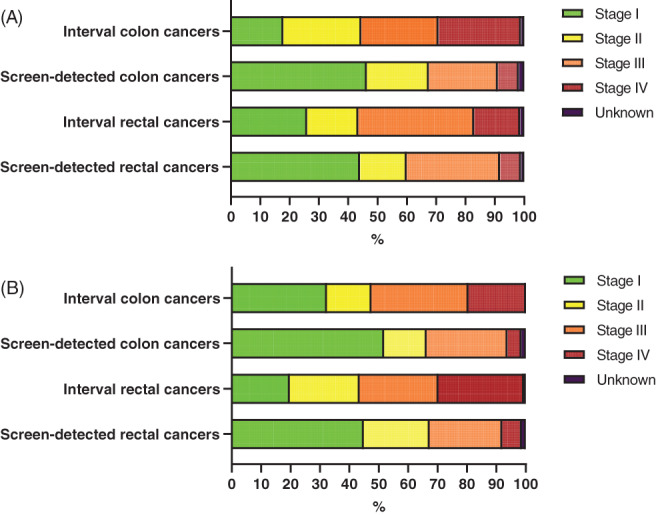
(A) Stage distribution interval and screening‐detected cancers after the first round. (B) Stage distribution interval and screening‐detected cancers after the second round

**FIGURE 3:**
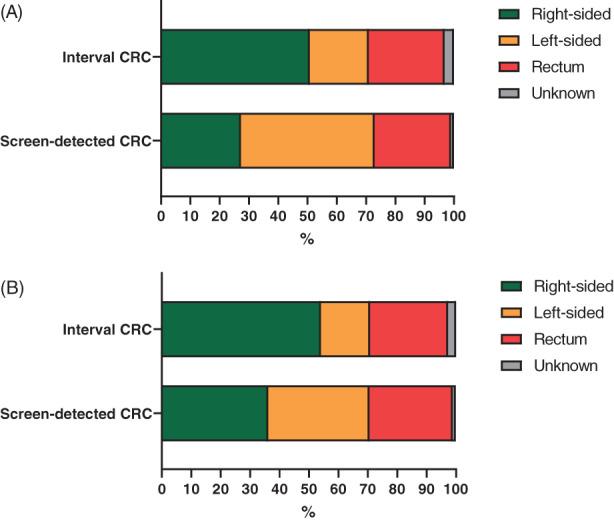
(A) Localization interval and screening‐detected cancers after the first round. (B) Localization interval and screening‐detected cancers after the second round

After both the first and second round, the stage distribution of interval colon cancers was less favorable than that of the screening‐detected colon cancers (*P* < .0001, Figure 2A). After the first round, 17.9% of interval colon cancers were assigned stage I, compared with 46.3% of screening‐detected colon cancers. By contrast, 28.1% of interval colon cancers were assigned stage IV, compared with 7.2% of screening‐detected colon cancers. The same pattern was observed after the second round (Figure 2B). In both rounds, interval colon cancers were more often located right‐sided than were the screening‐detected colon cancers (50.8% vs 27.3% in the first round and 54.1% vs 36.2% in the second round; *P* < .0001, Figure 3A, B).

After both the first and second round, the stage distribution of interval rectal cancers differed from that of screening‐detected rectal cancers (*P* < .0001, Figure 2A, B). After the second round, 26.0% of interval rectal cancers were assigned stage I, vs 44.0% of screening‐detected rectal cancers. By contrast, 15.7% of interval rectal cancers were assigned stage IV, vs 7.2% of screening‐detected rectal cancers. The proportions of cancers diagnosed in the rectum were quite comparable between interval and screening‐detected cancers, both in the first round (25.9% vs 26.1%, respectively) and in the second round (26.5% vs 28.3%, respectively; Figure 3A, B).

### 3.2 Association between f‐Hb concentration and interval CRC after the first round

**FIGURE 4:**
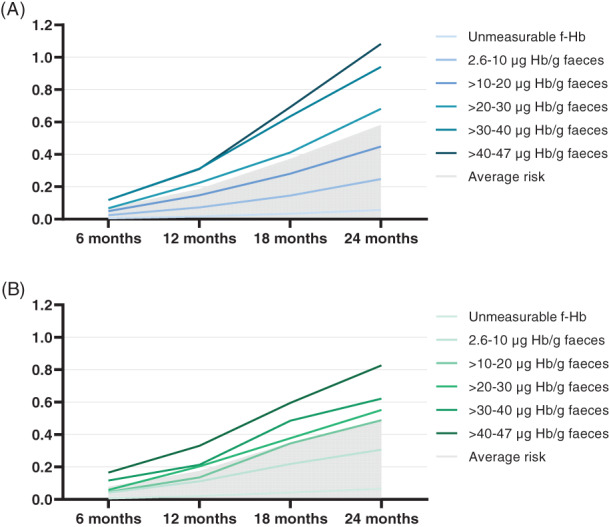
(A) Probability of detecting interval CRCs after the first round by subgroups of f‐Hb concentrations. (B) Probability of detecting interval CRCs after the second round by subgroups of f‐Hb concentrations

The vast majority (88.7%) of participants with a negative FIT had an unmeasurable f‐Hb concentration after the first round (Table 1). With increasing f‐Hb concentrations, the corresponding percentage of participants decreased. The probability of detecting an interval CRC increased with increasing f‐Hb concentrations and during the period until the next invitation after 24 months (Figure 4A). In participants with the highest f‐Hb concentration just below cut‐off (>40‐46.9 μg Hb/g feces), 1.08% had an interval CRC detected at 24 months, as opposed to 0.06% in those with an unmeasurable f‐Hb concentration (Figure 4A). After the first round, participants in the category with the highest f‐Hb concentrations (>40‐46.9 μg Hb/g feces) had an OR of 16.9 (95% CI 13.9‐20.3) for detection of interval CRC compared with participants with unmeasurable f‐Hb concentration, when adjusted for sex‐ and age‐differences (Model 1; Table 3).

**TABLE 3:** Multivariable logistic regression analysis: association between f‐Hb concentration and interval CRC in the first and second round, adjusted for sex‐ and age‐differences

	First screening round (Model 1) odds ratio, 95% CI	Second screening round (Model 2) odds ratio, 95% CI
Sex		
Men	REF	REF
Women	0.9 (0.9‐1.0)	0.8 (0.7‐1.0)
Age category*		
56‐60	REF	—
60‐64	1.8 (1.5‐2.2)	REF
65‐69	2.4 (2.0‐2.9)	1.6 (1.2‐2.1)
70‐74	3.8 (3.0‐4.7)	1.8 (1.3‐2.6)
≥75	4.3 (4.6‐5.3)	—
Prior f‐Hb concentration (μg Hb/g feces)*		
Unmeasurable (0‐2.6)	REF	REF
>2.6‐10	4.0 (3.5‐4.5)	4.7 (3.6‐6.0)
>10‐20	7.2 (6.3‐8.1)	7.2 (5.5‐9.3)
>20‐30	11.1 (9.5‐12.9)	8.2 (5.8‐11.2)
>30‐40	14.9 (12.7‐17.4)	9.9 (6.9‐13.7)
>40‐46.9	16.9 (13.9‐20.3)	12.0 (7.8‐17.6)

Abbreviations: 95% CI, 95% confidence interval; CRC, colorectal cancer; f‐Hb, fecal hemoglobin.

* *P* < .05.

### 3.3 Association between f‐Hb concentration and interval CRC after the second round

After the second round, again, most participants with a negative FIT had an unmeasurable f‐Hb concentration (92.9%, Table 1). The probability of detecting an interval CRC increased with higher f‐Hb concentrations and during the period until the next invitation (Figure 4B). In participants with the highest f‐Hb concentration just below cut‐off (>40‐46.9 μg Hb/g feces), 0.83% had an interval CRC detected at 24 months, as opposed to 0.07% in participants with unmeasurable f‐Hb concentrations (Figure 4B).

Similar to the first round, multivariable analysis showed a strong correlation between f‐Hb concentration and detection of interval CRC after the second round, when adjusted for sex‐ and age‐differences. Participants with the highest f‐Hb concentrations (>40‐46.9 μg Hb/g feces) had an OR of 12.0 (95% CI 7.8‐17.6) for detection of interval CRC compared with participants with unmeasurable f‐Hb concentrations (Model 2; Table 3).

Lastly, we compared different models for estimating the probability of detecting an interval CRC after the second round. These models were a variation of model 2 and took into account f‐Hb concentrations of the first round as well. Model 3a included dichotomous f‐Hb concentrations of the first round and categorical f‐Hb concentrations of the second round (AIC: 10236.53, Supplementary Table 3). Model 3b included summed f‐Hb concentrations of both rounds, dividing this added value into quantiles (AIC: 10268.59, Supplementary Table 4). The model that discriminated best was the one that included categorical f‐Hb concentrations of the first and second round separately (Model 3c, AIC: 10232.83, Table 4).

**TABLE 4:** Multivariable logistic regression analysis: association between f‐Hb concentrations of the first and second round and interval CRC in the second round, adjusted for sex‐ and age‐differences

	Second screening round (model 3c) odds ratio, 95% CI
Sex	
Men	REF
Women	0.9 (0.7‐1.0)
Age category	
56‐60	—
60‐64	REF
65‐69	1.5 (1.2‐2.1)
70‐74	1.7 (1.2‐2.5)
≥75	—
f‐Hb concentration first round (μg Hb/g feces)	
Unmeasurable (0‐2.6)	REF
>2.6‐10	1.5 (1.2‐1.8)
>10‐20	2.1 (1.6‐2.8)
>20‐30	2.3 (1.5‐3.4)
>30‐40	3.0 (1.8‐4.5)
>40‐46.9	1.7 (0.7‐3.4)
f‐Hb concentration second round (μg Hb/g feces)	
Unmeasurable (0‐2.6)	REF
>2.6‐10	3.9 (3.0‐5.0)
>10‐20	5.8 (4.4‐7.5)
>20‐30	6.4 (4.5‐8.9)
>30‐40	7.8 (5.4‐10.9)
>40‐46.9	9.3 (5.9‐13.7)

Abbreviations: 95% CI, 95% confidence interval; f‐Hb, fecal hemoglobin.

This model performed better than the model taking into account only the f‐Hb concentration measured in the second round (AIC: 10275.10). Thus, the goodness‐of‐fit of the model incorporating f‐Hb concentrations of two consecutive rounds (model 3c) was superior to the goodness‐of‐fit of the model only incorporating the last measured f‐Hb concentration (model 2).

## 4 DISCUSSION

This study evaluated the incidence of interval CRC and sensitivity of FIT after the first and the second screening round of the Dutch national FIT‐based CRC screening program. In both rounds, the incidence of interval CRC was low, whereas the sensitivity of FIT was high. Compared with screening‐detected CRC, interval CRC was more often diagnosed in men, more often at an advanced stage, and was more often located at the right side of the colon. Importantly, the higher the f‐Hb concentration, the higher the odds of detection of interval CRC, both after the first and the second round. The goodness‐of‐fit of the used model increased when f‐Hb concentrations of both rounds (as opposed to only the last measured f‐Hb concentration) were included to estimate the OR of interval CRC after the second round. This would suggest that not only the last measured f‐Hb concentration but also the prior screening history might be predictive for the detection of interval CRC.

Our results showed a high sensitivity of FIT in the Dutch CRC screening program. A systematic review on FIT‐sensitivity found a pooled sensitivity of FIT for CRC of 0.71 (95%CI 0.56‐0.83) in 12 studies that used a positivity cut‐off for FIT of >20 μg Hb/g feces.^14^ The measured FIT‐sensitivity in our study was slightly higher, but from that review it was not clear which round was assessed in the various studies. Furthermore, the sensitivity of FIT was calculated with a screening colonoscopy as the gold standard (ie, reference), whereas we have approximated the sensitivity from the interval CRC rate. The latter approach could result, however, in an over‐ or underestimation of the actual FIT‐sensitivity. Overestimation might occur when prevalent early‐stage CRCs went unrecognized as interval CRCs during the relevant time period. Underestimation might occur when interval CRCs actually were advanced adenomas at the time of prior FIT, which also impacts sensitivity estimates.

We approximated the FIT‐sensitivity in two ways: with the detection method and the proportional incidence method. The decrease in sensitivity over two rounds found with the detection method can be explained by the first round being a prevalence round, and subsequent rounds are incidence rounds. The sensitivity was estimated by dividing the number of screening‐detected CRCs by the sum of interval CRCs and screening‐detected CRCs. In the first round, prevalent cancers will most likely be detected through screening. Because most of the prevalent cancers will be diagnosed after the first round and the number of interval cancers detected will remain stable, we might expect a *plateau phase* in sensitivity of FIT after multiple screening rounds. This phenomenon has been described in several previous studies.^9^, ^15^, ^16^

The proportional incidence method allows for comparisons between programs, as it makes use of data on the (expected) background incidence of CRC in the target population. Moreover, the resulting estimate is unaffected by the effect of overdiagnosis. A very important caveat when calculating expected trends based on the CRC incidence in the pre‐screening era is that time trends cannot be taken into account. This phenomenon may lead to overestimation of the protective effect of the FIT. Still, our results testify to the satisfactory performance of the FIT in the Dutch CRC screening program. When calculating the sensitivity of FIT in a CRC screening program, there are a few caveats worth mentioning. From a screening program perspective, estimating sensitivity per screening round ensures that we can obtain the relevant measure of FIT sensitivity: CRC detection before clinical manifestation. Nevertheless, from a screening participant's point of view, one could argue that individuals with a screen‐detected CRC at the second screening round and a negative FIT at the first screening round are false negative test results and that this should be taken into account when estimating the sensitivity of the FIT in the first screening round. However, it is unknown what percentage of these screen‐detected CRCs were actually missed cancers in earlier screening, since colonoscopy is not performed in FIT‐negative individuals. Furthermore, it is unclear what percentage of screen‐detected CRCs should be included in this calculation, as it is unlikely that early‐stage screen‐detected CRCs were missed CRCs in the previous screening round. When advanced‐stage screen‐detected CRCs in the subsequent round are included in the calculation, this would (somewhat) reduce the FIT sensitivity. The evaluation of FIT‐based screening programs does not yet take this phenomenon into account when estimating the sensitivity of FIT.^15^, ^17^, ^18^, ^19^, ^20^, ^21^ Cancer screening researchers should discuss and reach consensus on the calculation of FIT sensitivity, similar to the consensus statement on post‐colonoscopy cancers.^22^

The finding that interval CRCs were more often diagnosed at the right side of the colon seems to underline the hypothesis that the FIT‐sensitivity is higher for left‐sided cancers and that right‐sided lesions are more frequently missed by FIT. A reason for this could be that approximately 75% of advanced serrated lesions are right‐sided, and tend to bleed less than do (advanced) adenomas. Furthermore, they are hypothesized to progress much faster into carcinoma than do adenomas once dysplasia has established.^23^, ^24^ A second hypothesis could be the degradation of hemoglobin, which may occur at a greater extent in right‐sided lesions, leading to lower concentrations of fecal hemoglobin. Unexpectedly, in the present study the proportion of rectal cancers diagnosed was similar for interval and screening‐detected cancers. Further research is necessary to find the reason for these missed rectal cancers.

Previous f‐Hb concentrations appear to have a greater predictive value for developing AN in future rounds than, for example, age, lifestyle or family history.^4^, ^25^, ^26^, ^27^ In this study, we used different models to estimate the probability of detecting an interval CRC after both rounds. We found that the model that incorporated f‐Hb concentrations of both the first and second round performed better to estimate probability of detecting an interval CRC after the second round than did the model that included only the last measured f‐Hb concentration after the second round. This indeed goes to show that prior screening history could be predictive for detection of interval CRC. When we assessed the predictive value of the variation in both f‐Hb concentrations (ie, the *delta*) on the probability of detecting interval CRCs, this model was not significant. We expected a higher association between this *delta* and detection of interval CRC after the second round. However, when information on CRCs of multiple screening rounds becomes available, the prior screening history—that is, the variation in f‐Hb concentrations—could allow identifying individuals at highest probability of detecting an interval CRC with the use of more advanced statistics such as a (linear) mixed model.

Although the incidence of interval CRC was low after both rounds, the largest proportion of interval CRCs was diagnosed at an advanced stage. As these are associated with higher morbidity and mortality, the importance of preventing these interval CRCs is self‐evident. Of note, we found substantial differences in the probability of detecting an interval CRC by f‐Hb concentration, like in recent studies from Spain and Italy.^8^, ^28^ There are several options to address participants at highest probability of developing an interval CRC, hereby increasing benefits of the screening program. In case of a history of multiple previous f‐Hb concentrations just below the cut‐off, they can be offered colonoscopy. Alternatively, the screening interval can be shortened, thereby intensifying FIT‐based screening. Clearly, the first option would require additional colonoscopy capacity. In our study, this would require approximately 10% additional colonoscopy capacity per screening round. Both options warrant close consultation with public health officials, while considering that information on multiple screening rounds should be available to make well‐balanced decisions on these strategies, especially with intensifying FIT‐screening. In the Netherlands, every year approximately two million individuals were invited to participate in the screening program, of whom about 72% participated.^29^ Around 95% of them had a negative FIT. In this study, we found that only 10% of all participants with a negative FIT had detectable f‐Hb concentrations below the cut‐off (>2.6‐47 μg Hb/g feces). Importantly, around 50% of all interval CRCs had been diagnosed in this small population. The associated higher probability of detecting an interval CRC in this small population, coupled with the large proportion of participants with a negative FIT *and* an unmeasurable f‐Hb concentration, indicates possibilities for risk‐stratified CRC screening. Such a program could improve the harm‐benefit balance, increase the yield of AN (in terms of detection rate and positive predictive value) and imply a lower burden of screening for participants at low risk. Still, factors such as acceptability, participation and use of resources need to be considered as well.^30^

We reported on probability of detecting interval CRCs for different categories of f‐Hb concentration, thus making these data generalizable to programs using other cut‐offs. Obviously, the generalizability is highly dependent on the set‐up of the program (ie, population‐based vs opportunistic screening). Another important strength of this study is the large sample size, enabling us to combine essential information on interval CRC in a national, organized screening program. The main limitation of this study is that we could incorporate only data from two rounds. This is due to a data acquisition delay of information on CRC, such as the stage distribution. We hope that after having analyzed information from multiple rounds of FIT screening we will be able to identify which and how patterns of f‐Hb concentrations influence the probability of detecting interval CRCs.

To conclude, we found that the CRC screening program in the Netherlands has a low incidence of interval CRC and an associated high FIT‐sensitivity, after one and two consecutive screening rounds. The probability of detecting interval CRCs increased with increasing fecal hemoglobin concentrations. Our findings suggest there is a potential for further optimizing CRC screening programs with the use of risk‐stratified CRC screening based on prior fecal hemoglobin concentrations.

## AUTHOR CONTRIBUTIONS

Emilie C. H. Breekveldt, Esther Toes‐Zoutendijk, Monique E. van Leerdam and Iris Lansdorp‐Vogelaar conceptualized the study and contributed to the study design. Emilie C. H. Breekveldt performed the analyses and accessed and verified the data. Emilie C. H. Breekveldt wrote the draft version of the manuscript, with supervision from Esther Toes‐Zoutendijk, Monique E. van Leerdam and Iris Lansdorp‐Vogelaar. All authors contributed to reviewing drafts of the manuscript and approved the final manuscript draft. The work reported in the paper has been performed by the authors, unless clearly specified in the text.

## CONFLICT OF INTEREST

Manon C. W. Spaander receives financial research support from Sysmex/Sentinel; FIT tubes and analyses for research are provided. All other authors declare no conflicts of interest.

## ETHICS STATEMENT

This study was conducted in accordance with the Dutch population screening act (WBO). Returning the FIT is considered as consent for using pseudonymised data of all screening colonoscopy reports, following the WBO. All individuals had the right to object to the use of their data.

## DISCLAIMER

This analysis has been carried out as part of the national monitoring and evaluation of the colorectal cancer screening program, funded by the National Institute for Public Health and the Environment (RIVM). The funder had no role in the study design, data analysis and preparation of the manuscript.

## Supporting information


**SUPPLEMENTARY TABLE 1** Incidence of interval CRC after negative FIT and sensitivity of FIT after the first screening round
**SUPPLEMENTARY TABLE 2**. Incidence of interval CRC after negative FIT and sensitivity of FIT after the second screening round
**SUPPLEMENTARY TABLE 3**. Multivariable logistic regression analysis: association between dichotomous f‐Hb concentrations in the first screening round, f‐Hb concentration in the second round and interval CRC in the second screening round, adjusted for sex‐ and age‐differences (Model 3a)
**SUPPLEMENTARY TABLE 4**. Multivariable logistic regression analysis: association between summed f‐Hb concentrations from the first and second round (quantiles), and interval CRC in the second screening round, adjusted for sex‐ and age‐differences (Model 3b)

## Data Availability

The data that support the findings of this study are on request available from the last author.
