# Supplementary material for: Factors associated with interval colorectal cancer after negative FIT: Results of two screening rounds in the Dutch FIT‐based CRC screening program
Source: Int J Cancer. 2022 Dec 1;152(8):1536–46. doi: 10.1002/ijc.34373 (PMC10107864; doi:10.1002/ijc.34373)
Supplement: Supplementary file 1 — SUPPLEMENTARY TABLE 1 Incidence of interval CRC after negative FIT and sensitivity of FIT after the first screening round SUPPLEMENTARY TABLE 2. Incidence of interval CRC after negative FIT and sensitivity of FIT after the second screening round SUPPLEMENTARY TABLE 3. Multivariable logistic regression analysis: association between dichotomous f‐Hb concentrations in the first screening round, f‐Hb concentration in the second round and interval CRC in the second screening round, adjusted for sex‐ and age‐differences (Model 3a) SUPPLEMENTARY TABLE 4. Multivariable logistic regression analysis: association between summed f‐Hb concentrations from the first and second round (quantiles), and interval CRC in the second screening round, adjusted for sex‐ and age‐differences (Model 3b) [file IJC-152-1536-s001.pdf]

Factors Associated With Interval Colorectal Cancer after Negative FIT: Results of Two Screening Rounds in the Dutch FIT-based CRC Screening Program

Emilie C. H. Breekveldt, Esther Toes-Zoutendijk, Hilliene J. van de Schootbrugge-Vandermeer, Lucie de Jonge, Arthur I. Kooyker, Manon C. W. Spaander, Anneke J. van Vuuren, Folkert J. van Kemenade, Christian Ramakers, Evelien Dekker, Iris D. Nagtegaal, Monique E. van Leerdam, and Iris Lansdorp-Vogelaar.

Supplementary materials – Table of contents

1. Supplementary Table 1. Incidence of interval CRC after negative FIT and sensitivity of FIT after the first screening round
2. Supplementary Table 2. Incidence of interval CRC after negative FIT and sensitivity of FIT after the second screening round
3. Supplementary Table 3. Multivariable logistic regression analysis: association between dichotomous f-Hb concentrations in the first screening round, f-Hb concentration in the second round, and interval CRC in the 2nd screening round, adjusted for sex- and age-differences (Model 3a)
4. Supplementary Table 4. Multivariable logistic regression analysis: association between summed f-Hb concentrations from the first and second round (quantiles), and interval CRC in the 2nd screening round, adjusted for sex- and age-differences (Model 3b)

Supplementary Table 1. Incidence of interval CRC after negative FIT and sensitivity of FIT after the first screening round

		NUMBER			INCIDENCE RATE/10,000			RR	SENSITIVITY (DETECTION METHOD) (%, 95%CI)	SENSITIVITY (PI METHOD) (%, 95%CI)
		POPULATION SCREENED	IC	SDC	IC	SDC	CRC PREDICTED*	IC	SDC/SDC + IC	1-RR
**YEAR**	**SEX**									
	**Male**	**1,113,736**	**1,178**	**7,584**	**10.6**	**68.1**	**50.6**	**0.21**	**86.6 (85.8-87.3)**	**79.0 (74.7-83.7)**
2014		149,000	173	1,184	11.6	79.5	61.4	0.19	87.3 (85.5-89.0)	81.1 (69.9-94.1)
2015		392,748	442	2,813	11.3	71.6	51.8	0.22	86.4 (85.2-87.6)	78.2 (71.2-85.8)
2016		335,548	340	2,268	10.1	67.6	49.2	0.21	87.0 (85.7-88.3)	79.5 (71.5-88.4)
2017		236,440	223	1,319	9.4	55.8	43.7	0.22	85.5 (83.8-87.3)	78.5 (68.8-89.5)
	**Female**	**1,188,975**	**1,078**	**4,599**	**9.1**	**38.7**	**33.1**	**0.28**	**81.0 (80.0-82.0)**	**72.5 (68.3-77.0)**
2014		155,816	192	705	12.3	45.2	40.1	0.31	78.6 (75.9-81.3)	69.4 (60.2-79.9)
2015		415,218	400	1,704	9.6	41.0	33.9	0.28	81.0 (79.3-82.7)	71.7 (65.0-79.1)
2016		359,594	333	1,357	9.3	37.7	32.4	0.29	80.3 (78.4-82.2)	71.3 (64.0-79.4)
2017		258,347	163	833	6.3	32.2	28.5	0.22	83.6 (81.3-85.9)	77.9 (66.8-90.8)
	**AGE (YRS)**									
	**55-59**	**353,178**	**122**	**899**	**3.5**	**25.5**	**17.4**	**0.20**	**88.1 (86.1-90.0)**	**79.9 (66.9-95.4)**
2014**										
2015**										
2016		131,000	54	351	4.1	26.8	17.5	0.23	86.7 (83.4-90.0)	76.6 (58.6-100.0)
2017		222,177	68	548	3.1	24.7	17.3	0.18	89.0 (86.5-91.4)	82.1 (64.7-104.1)
	**60-64**	**813,106**	**594**	**3,248**	**7.3**	**40.0**	**29.5**	**0.25**	**84.5 (83.4-85.7)**	**75.3 (69.4-81.6)**
2014		65,329	54	289	8.3	44.2	29.8	0.28	84.3 (80.4-88.1)	72.1 (55.2-94.2)
2015		307,754	219	1,298	7.1	42.2	29.6	0.24	85.6 (83.8-87.3)	76.0 (66.5-86.7)
2016		306,822	242	1,224	7.9	39.9	29.4	0.27	83.5 (81.6-85.4)	73.2 (64.5-83.0)
2017		133,201	79	437	5.9	32.8	29.3	0.20	84.7 (81.6-87.8)	79.9 (64.1-99.6)
	**65-69**	**673,110**	**729**	**3,985**	**10.8**	**59.2**	**46.1**	**0.23**	**84.5 (83.5-85.6)**	**76.6 (71.2-82.3)**
2014		174,659	177	989	10.1	56.6	46.2	0.22	84.8 (82.8-86.9)	78.2 (67.4-90.6)
2015		434,841	488	2,580	11.2	59.3	46.1	0.24	84.1 (82.8-85.4)	75.7 (69.3-82.7)
2016		60,976	63	404	10.3	66.3	45.9	0.22	86.5 (83.4-89.6)	77.6 (60.6-99.3)
2017**										
	**70-74**	**187,583**	**279**	**1,511**	**14.9**	**80.6**	**62.9**	**0.24**	**84.4 (82.7-86.1)**	**76.3 (67.9-85.8)**
2014**										
2015**										
2016		110,092	159	886	14.4	80.5	63.0	0.23	84.8 (82.6-87.0)	77.1 (66.0-90.1)
2017		77,490	120	625	15.5	80.7	62.9	0.25	83.9 (81.3-86.5)	75.4 (63.0-90.1)
	**≥75**	**275,734**	**532**	**2,540**	**19.3**	**92.1**	**83.5**	**0.23**	**82.7 (81.3-84.0)**	**76.9 (70.6-83.7)**
2014		64,828	124	611	18.1	94.2	82.7	0.22	83.1 (80.4-85.8)	78.1 (65.5-93.2)
2015		65,369	135	639	20.7	97.8	83.3	0.25	82.6 (79.9-85.2)	75.2 (63.5-89.0)
2016		86,252	155	760	18.0	88.1	83.9	0.21	83.1 (80.6-85.5)	78.5 (67.1-92.0)
2017		59,285	118	530	19.9	89.4	84.0	0.24	81.8 (78.8-84.8)	76.3 (63.7-91.4)
	**TOTAL**	**2,302,711**	**2,256**	**12,183**	**9.8**	**52.9**	**41.6**	**0.24**	**84.4 (83.8-85.0)**	**76.4 (73.3-79.6)**

Abbreviations: IC: interval colorectal cancer. SDC: screening-detected colorectal cancer. CRC: colorectal cancer. RR: rate ratio. CI: confidence interval. PI: proportional incidence. Yrs: years.

* based on expected CRC incidence using Poisson log linear regression to extrapolate CRC incidence data from the pre-screening era. Displayed for the screening interval of 1.97 years in the first round and 1.96 years in the second round.

**too few people screened/too few cancers for displaying/significance.

Supplementary Table 2. Incidence of interval CRC after negative FIT and sensitivity of FIT after the second screening round

		NUMBER			INCIDENCE RATE/10,000			RR	SENSITIVITY (DETECTION METHOD) (%, 95%CI)	SENSITIVITY (PI METHOD) (%, 95%CI)
		POPULATION SCREENED	IC	SDC	IC	SDC	CRC PREDICTED*	IC	SDC/SDC + IC	1-RR
**YEAR**	**SEX**									
	**Male**	**334,559**	**366**	**1,066**	**10.9**	**31.9**	**56.6**	**0.19**	**74.4 (72.2-76.7)**	**80.7 (72.9-89.4)**
2016		98,273	125	332	12.7	33.8	55.5	0.23	72.6 (68.6-76.7)	77.1 (64.7-91.9)
2017		236,286	241	734	10.2	31.1	57.0	0.18	75.3 (72.6-78.0)	82.1 (72.4-93.2)
	**Female**	**369,336**	**299**	**808**	**8.1**	**21.9**	**36.1**	**0.22**	**73.0 (70.4-75.6)**	**77.5 (69.2-86.8)**
2016		105,951	93	220	8.8	20.8	36.0	0.24	70.3 (65.2-75.4)	75.5 (61.6-92.6)
2017		263,385	216	588	8.2	22.3	36.1	0.23	73.1 (70.1-76.2)	77.3 (67.6-88.3)
	**AGE (YRS)**									
	**60-64**	**76,542**	**46**	**143**	**6.0**	**18.7**	**29.1**	**0.21**	**75.7 (69.5-81.8)**	**79.4 (59.4-106.0)**
2016**										
2017		74,517	44	142	5.9	19.1	29.1	0.20	76.3 (70.2-82.5)	79.7 (59.3-107.1)
	**65-69**	**532,388**	**519**	**1,416**	**9.7**	**26.6**	**45.5**	**0.21**	**73.2 (71.2-75.2)**	**78.7 (72.2-85.7)**
2016		202,190	216	551	10.7	27.3	45.7	0.23	71.8 (68.7-75.0)	76.6 (67.0-87.5)
2017		330,198	303	865	9.2	26.2	45.4	0.20	74.1 (71.5-76.6)	79.7 (71.2-89.2)
	**≥70**	**94,964**	**110**	**315**	**11.6**	**33.2**	**62.3**	**0.19**	**74.1 (70.0-78.3)**	**81.4 (67.5-98.1)**
2016**										
2017		94,955	110	315	11.6	33.2	62.3	0.19	74.1 (70.0-78.3)	81.4 (67.5-98.1)
	**TOTAL**	**703,895**	**675**	**1,874**	**9.6**	**26.6**	**45.9**	**0.21**	**73.5 (71.8-75.2)**	**79.1 (73.3-85.3)**

Abbreviations: IC: interval colorectal cancer. SDC: screen-detected colorectal cancer. CRC: colorectal cancer. RR: rate ratio. CI: confidence interval. PI: proportional incidence. Yrs: years.

* based on expected CRC incidence using Poisson log linear regression to extrapolate CRC incidence data from the pre-screening era. Displayed for the screening interval of 1.97 years in the first round and 1.96 years in the second round.

**too few people screened/too few cancers for displaying/significance.

Supplementary Table 3. Multivariable logistic regression analysis: association between dichotomous f-Hb concentrations in the first screening round, f-Hb concentration in the second round, and interval CRC in the 2nd screening round, adjusted for sex- and age-differences (Model 3a)

	Odds Ratio, 95% CI
**Sex**	
Men	REF
Women	0.9(0.7-1.0)
**Age category**	
60-64	REF
65-69	1.5(1.2-2.1)
≥70	1.8(1.3-2.5)
**F-Hb concentration round 1 (μg Hb/g feces)**	
Unmeasurable (0-2.6)	REF
>2.6-46.9	1.8 (1.5-2.1)
**F-Hb concentration round 2 (μg Hb/g feces)**	
Unmeasurable (0-2.6)	REF
>2.6-10	3.9(3.0-5.1)
>10-20	6.0(4.5-7.7)
>20-30	6.7(4.7-9.3)
>30-40	8.1 (5.6-11.3)
>40-46.9	9.7(6.3-14.4)

Abbreviations: 95% CI = 95% Confidence interval. f-Hb = fecal hemoglobin.

Supplementary Table 4. Multivariable logistic regression analysis: association between summed f-Hb concentrations from the first and second round (quantiles), and interval CRC in the 2nd screening round, adjusted for sex- and age-differences (Model 3b)

	Odds Ratio, 95% CI
**Sex**	
Men	REF
Women	0.9 (0.8-1.0)
**Age category**	
60-64	REF
65-69	1.4 (1.2-1.7)
≥70	1.5 (1.1-1.9)
**Summed f-Hb concentration round 1 + round 2, quantiles (µg Hb/g feces)**	
Unmeasurable (0-2.6)	REF
1st quantile	1.9 (1.4-2.7)
2nd quantile	2.7 (2.0-3.6)
3rd quantile	5.2 (4.2-6.5)
4rd quantile	8.3 (6.9-10.0)

Abbreviations: 95% CI = 95% Confidence interval. f-Hb = fecal hemoglobin.
